# Effects of Client-Centered Occupational Therapy Intervention in Older Adults With Depression: A Randomized Controlled Trial

**DOI:** 10.1155/oti/5559899

**Published:** 2025-02-18

**Authors:** HyeongGi Jeong, DeokJu Kim

**Affiliations:** Department of Occupational Therapy, College of Health & Medical Sciences, Cheongju University, Cheongju, Republic of Korea

**Keywords:** client-centered occupational therapy, community participation, depression, mental health, older adults

## Abstract

**Objective:** The purpose of this study was to determine the impact of client-centered occupational therapy intervention on the mental health, activities of daily living (ADL), and community participation of older adults with depression.

**Methods:** This study was a single-blind, randomized controlled study conducted on older adults with depression, and those who met the selection and exclusion criteria were randomly divided into an experimental group of 15 people and a control group of 15 people. The experimental group performed client-centered occupational therapy intervention using the Canadian Occupational Performance Measure (COPM) and Barth Time Construction (BTC), and the control group performed case management and a strength-brain exercise program. Both groups underwent intervention twice a week for 60 min, for a total of 16 sessions. Before and after the intervention, depression, anxiety, stress, ADL, and community partitioning were measured using assessment tools with proven reliability and validity in both the experimental group and the control group.

**Results:** The experimental group exhibited significant changes after the intervention in depression, anxiety, stress, social integration, and community integration scores (*p* < 0.05 and *p* < 0.01), while the control group showed significant changes only in depression scores (*p* < 0.05). As a result of comparing the amount of change between the experimental and control groups, significant differences were found in social integration among the areas of depression, stress, and community integration (*p* < 0.05 and *p* < 0.01).

**Conclusion:** These results suggest that client-centered occupational therapy intervention could serve as an effective intervention for improving mental health, ADL, and community participation in older adults with depression.

**Trial Registration:** Korea Clinical Research Information Service (CRIS): KCT0009358

## 1. Introduction

As of 2022, the older adult population (≥ 65 years) accounts for 17.5% of the total population in South Korea and is projected to increase to 20.6% by 2025, entering into a super-aged society [1]. This rapid aging has brought various issues such as an increase in older adults suffering from chronic diseases, feelings of loss, and depression due to the death of a spouse or friends [2]. Older adults are typically marked by overall health issues, such as functional loss and chronic diseases, due to physical changes [3]. Additionally, psychosocial difficulties can emerge due to economic poverty and social isolation, making older adults more susceptible to depression [4]. According to a survey by the Ministry of Health and Welfare, 13.5% of the entire older adult population in Korea is reported to have depressive symptoms, highlighting the need for social attention towards depression in older adults [5].

Depression in the older adults often progresses latently compared to other age groups, leading to a tendency to dismiss it as a natural part of aging, which frequently results in worsening symptoms [6]. Considering the gradual entry into a super-aged society, the number of older adults with depression is expected to increase further [7]. Anxiety and stress are other common mental health issues in older adults, exacerbating their difficulties with social integration when present to a significant degree [8]. Effectively managing these conditions is crucial, as they frequently stem from challenges in adapting to new changes, in addition to declines in cognitive and physical functions [9, 10].

To support older adults in leading a healthy life, it is of crucial importance to facilitate their engagement in activities of daily living (ADL) and active participation in community activities. A decline in ADL abilities due to aging may hinder older adults' independence, necessitating assistance from family or others. Limited engagement in social activities can significantly impair the quality of life for the older adults. Moreover, the absence of structured daily routines often leads to adverse emotional outcomes, exacerbating feelings of anxiety, depression, and social isolation [11]. Structured daily lives can enhance self-regulation in individuals with depression, underscoring the importance of establishing and adhering to schedules for ADL [12]. Consequently, improving the older adults' capacity to perform ADL is essential for fostering a healthy lifestyle, satisfaction, and successful aging, and structured daily routines and increased activity levels contribute to maintaining independent living [13]. Research by Kang and Park [14] demonstrated that increased involvement in community activities lowers depression levels in both urban and rural older adult populations. Likewise, Gweon [15] found that community participation markedly reduces depression, anxiety, and stress among the older adults, affirming that active engagement with their social environment can significantly enhance their mental health.

As previously mentioned, depression is a critical psychiatric condition that is closely associated with the daily lives of older adults, who are particularly vulnerable to health issues. To counter the negative impact of depression on the physical, psychological, and social functions necessary for living in a community, it is essential to provide older adults who are suffering from depression with active support through various interventions to facilitate recovery. Client-centered occupational therapy is a top-down approach widely used in clinical settings, where goals are established based on occupational performance and personalized training is provided by an occupational therapist, ensuring a holistic approach to therapy that is tailored to each patient's unique needs and circumstances [16, 17]. Numerous studies have shown that this intervention positively influences clients' mental health, ADL, and quality of life [18–20].

The rapid aging process in South Korea highlights the critical need for research focusing on the mental health of the older adult population. Although client-centered occupational therapy has emerged as an intervention model that provides hope and motivation, its application to patients with depression remains unexplored, and little research has been dedicated to the community involvement of older adults with depression. In this context, this study is aimed at exploring the impact of client-centered occupational therapy on the mental health, ADL, and community participation of older adults suffering from depression. The hypothesis of this study is as follows. Client-centered occupational therapy will have a positive effect on lowering depression, anxiety, and stress in the older adults with depression and improving ADL and social participation ability.

## 2. Methods

### 2.1. Participants

This study was carried out from June to September 2023, involving 30 older adults (aged 65 and older) diagnosed with depression for 2 years or longer, registered at the O Mental Health Welfare Center in Chungcheongbuk-do. The inclusion criteria included the following: (1) diagnosis of depression (F30–F39) by a psychiatrist, (2) aged 65 or older, (3) diagnosis of chronic depression for two or more years, (4) under depression medication for two or more years, (5) understanding of the purpose of this study and consent to participate in the study, and (6) no communication impairments. The exclusion criteria encompassed the following: (1) presence of other neurological or surgical conditions, (2) recent participation in other research studies or drug trials, (3) mobility limitations preventing participation in community activities, and (4) visual field impairments.

### 2.2. Study Design

**Figure 1:**
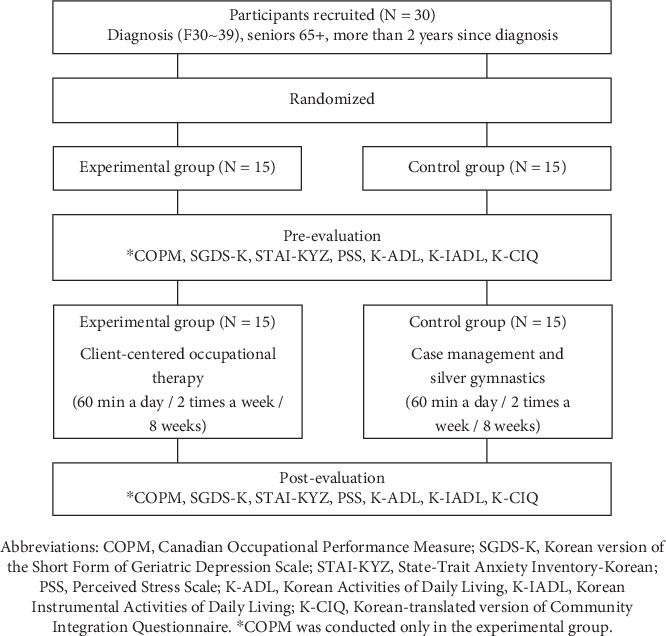
Study process.

This study was conducted as a single-blind randomized controlled trial. Before conducting the research, the ethical protection of the participants was ensured by securing preliminary approval from the Institutional Review Board (IRB) of Cheongju University (Approval Number 1041107-202306-HR-020-01). Sample size was measured through G-Power 3.0 software to recruit participants in this study. The effect size was set to 0.8, the signature level was set to 0.05, and the power was 80%, and the minimum number of people required for the study was calculated. Before participation, all individuals were thoroughly briefed on the study's aims and procedures and provided written consent. Thirty older adults with depression, meeting the specified inclusion criteria, were randomly allocated to either the experimental or control group via a computerized selection process. Before initiating the intervention, those in the experimental group selected activities they found personally meaningful with the assistance of the Canadian Occupational Performance Measure (COPM). They also completed the Barth Time Construction (BTC) to examine their daily routines and designate times for these activities. Interventions were conducted in participants' homes, with the experimental group receiving client-centered occupational therapy twice weekly for 60 min across 16 sessions and the control group receiving psychological stability-focused case management and silver gymnastics instruction, also twice weekly for 60 min over 16 sessions. Participants were blind to their group allocation. The study was facilitated by two occupational therapists, each with a minimum of 3 years of professional experience, and followed a structured timeline that included pre-evaluation, intervention, and postevaluation stages. For the control group's intervention, occupational therapists were trained with a brain-power gymnastics video from the National Health Insurance Service, focusing on the methodology and efficacy of the exercises. After the intervention, a meeting was held to go through case management logs and confirm the effective execution of the activity program (Figure 1).

### 2.3. Client-Centered Occupational Therapy Intervention

**Figure 2:**
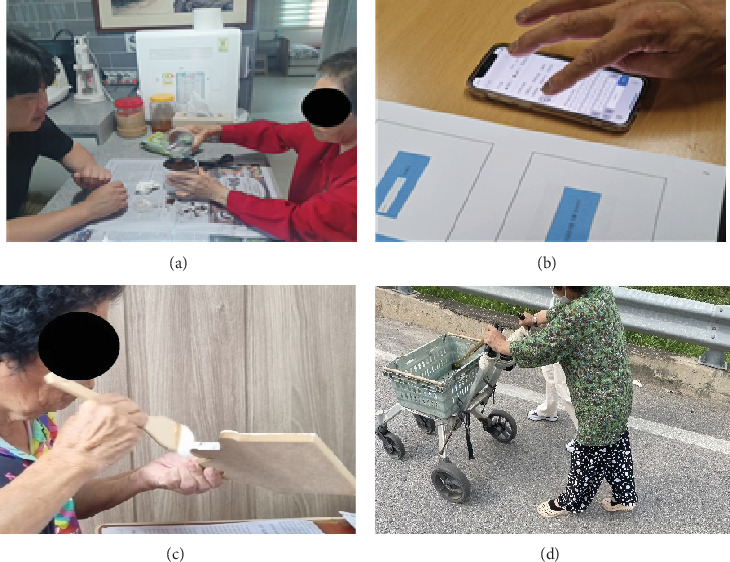
Client-centered occupational therapy intervention (tasks selected using COPM): (a) planting; (b) social media; (c) handicraft; (d) walking to the public health center and back.

Client-centered occupational therapy focuses on setting performance-based goals, with therapists offering direct guidance to clients on executing selected activities. This personalized approach considers the client's environment and occupation, ensuring that the therapy is tailored to their specific needs and circumstances. This approach employs the basic concept of occupational therapy designed to enable clients to actively adapt to their given situation by restoring necessary functions through meaningful and purposeful activities. In this study, we utilized the stages of the client-centered self-care intervention (CCSCI) developed by Guidetti et al. [21]. The CCSCI is a nine-stage intervention designed to empower clients in managing their occupational performance and problem-solving, guiding them through nine process steps from planning to evaluation, encapsulated within a goal–plan–check framework. We adapted the nine stages of CCSCI to craft the basic structure of the intervention program, tailoring them to meet the specific objectives of this study. The 16-session intervention program was structured as follows: Session 1 focused on establishing rapport and conducting a preliminary evaluation; Session 2 involved goal setting alongside activity observation and analysis; Session 3 was dedicated to planning for occupational performance; Sessions 4 through 15 involved the implementation of this plan; and Session 16 concluded with a postevaluation. Among the CCSCI stages, Stage 7, aimed at sharing goals and performance plans with other rehabilitation teams, was omitted due to its lack of applicability to our study's framework [22] (Tables 1 and 2) (Figure 2).

### 2.4. Case Management and Silver Gymnastics

The intervention for the control group was structured into two distinct parts: a 30-min case management consisting of counseling for psychological stability, medication management, and assistance with scheduling hospital visits, followed by a 30-min silver gymnastics [23], beginning with a seated stretching routine designed to relax tense muscles and enhance blood circulation, followed by the brain-power gymnastics aimed at activating the parasympathetic nervous system and fostering a stable emotional state. In this study, the 30-min silver gymnastics program was administered to the control group twice weekly over 8 weeks.

### 2.5. Measurements

#### 2.5.1. Occupational Performance

Participants' performance and satisfaction concerning their self-selected goals were evaluated before and after the intervention using the COPM. The COPM is a semistructured assessment tool designed to enable clients to identify tasks they want and need to perform, measuring outcomes in self-care, productivity, and leisure. Participants rate the importance of selected tasks and their performance and satisfaction on a 10-point rating scale ranging from 1 (*having great difficulty with performance*/*not at all satisfied*) to 10 (*no difficulty with performance*/*completely satisfied*), with higher scores indicating better performance and greater satisfaction. Test–retest reliability ranged from 0.65 to 0.80 for the performance scale and 0.75 to 0.89 for the satisfaction scale [24].

#### 2.5.2. Time Construction

The BTC was used to identify daily life patterns and select times for performing the selected tasks. The BTC is a variation of the configuration that relies less on reading ability and consists of 24 horizontal lines representing the hours of the day. It uses colors to distinguish 12 categories (e.g., blue for sleep, orange for eating, and black for watching TV). Participants fill in the timetable with colored pencils to indicate their daily activities. The total time for each activity is converted into a percentage of hours per week. Test–retest reliability was determined to be 0.95 [25].

#### 2.5.3. Depression

The level of depression in older adults was assessed using the Korean version of the Short Form of the Geriatric Depression Scale (SGDS-K). It consists of 15 items, and each item is scored on a binary scale of 1 (*yes*) and 0 (*no*), with some items reverse-scored (1 = *no*, 0 = *yes*). Higher scores indicate more severe symptoms of depression, with scores of 8 and above out of 15 points considered indicative of depression. Test–retest reliability was 0.75 [26, 27].

#### 2.5.4. Anxiety

The State–Trait Anxiety Inventory-Korean YZ (STAI-KYZ) was used to measure anxiety. This instrument assesses both state anxiety, a temporary emotional state subject to change over time, and trait anxiety, more stable individual differences unaffected by psychological stress. It comprises 20 items each for state and trait anxiety, scored on a 4-point Likert scale, with a total score ranging from 20 to 80, where a higher score indicates a higher level of anxiety. The internal reliability at the time of development was 0.88 [28].

#### 2.5.5. Stress

The level of stress was measured with the Perceived Stress Scale (PSS), which probes into stress-related experiences over the past month. The PSS comprises 10 items rated on a 5-point scale, with positive items reverse-scored, resulting in total scores that vary from 0 to 40. Higher scores indicate higher levels of perceived stress. The scale's internal reliability was determined to be 0.79, 0.87, and 0.84 for positive, negative, and overall perceptions of stress, respectively [29].

#### 2.5.6. ADL

The Korean Activities of Daily Living (K-ADL) [30] was used to assess basic ADL abilities among the participants, and the Korean Instrumental Activities of Daily Living (K-IADL) [31] was used to evaluate their instrumental activities of daily living (IADL) abilities. The K-ADL includes seven items covering basic functions such as dressing, washing, bathing, eating, moving, toileting, and bowel and bladder movements. The K-IADL consists of 10 items, including grooming, housekeeping, meal preparation, laundry, going out nearby, transportation, shopping, managing finances, using the telephone, and taking medication. Scoring is based on the frequency of activities performed in the most recent week, with higher scores reflecting an increased need for assistance in IADL.

#### 2.5.7. Social Participation

The Korean Community Integration Questionnaire (K-CIQ) is a 15-item questionnaire designed to assess community participation in three domains: home integration, social integration, and productive activity. Items 1–12 are rated on a 3-point scale, and Items 13 to 15 on a 6-point scale are combined into a single composite variable according to a scoring table. A total score ranges from 0 to 29 points, with higher scores indicating greater independence and community integration. Test–retest reliability of this instrument was 0.94 for home integration, 0.92 for social integration, and 0.99 for productive activity [32].

### 2.6. Data Analysis

All statistical analyses in this study were performed using SPSS version 27.0. Shapiro–Wilk test was conducted to test the normality of the two groups, and nonparametric analysis was performed because the normality was not satisfied. Among the general characteristics, mean (SD) was obtained for age and education level, and percentage for each item was obtained for the other items. To verify the homogeneity between the two groups, categorical variables such as gender, occupation, and living arrangement were analyzed using *χ*^2^ test; among the response items, if the expected frequency of cells lower than 5 exceeded 20%, the analysis was performed using Fisher's exact test. Other general characteristics and continuous categories such as assessment score were analyzed using the Mann–Whitney *U* test. Within-group differences between baseline and postintervention were analyzed using the Wilcoxon signed-rank test, and between-group differences between the two groups before and after the intervention were examined using the Mann–Whitney *U* test. The statistical significance level for all data was set at 0.05.

## 3. Results

### 3.1. Demographics and Baseline Characteristics

The general characteristics of the participants are outlined as follows: The average age was 75.07 ± 8.18 years in the experimental group and 75.80 ± 6.37 years in the control group. The experimental group consisted of 6 males (40.0%) and 9 females (60.0%), and the control group 4 males (26.7%) and 11 females (73.3%). The most prevalent educational achievement in both groups was high school graduation (*n* = 6 for each, 40.0%). Occupation-wise, the experimental group predominantly included unemployed individuals (*n* = 11, 73.3%), followed by those involved in agriculture (*n* = 2, 13.3%) and sales/service sector (*n* = 2, 13.3%). In the control group, there were 9 (60.0%) unemployed participants, 3 (20.0%) in agriculture, 2 (13.3%) in sales/service, and 1 (6.7%) participating in the senior employment scheme. The most common living arrangement for both groups was living alone. Apart from the K-CIQ domain “productive activity,” no significant differences were found in the general characteristics or all baseline scores between the two groups (*p* > 0.05) (Table 3).

### 3.2. Depression, Anxiety, and Stress

The analysis of mental health outcomes before and after the intervention for both groups indicated statistically significant improvements in the SGDS-K, STAI-KYZ, and PSS scores in the experimental group postintervention, compared to their baseline scores (*p* < 0.05 and *p* < 0.01). Conversely, the control group exhibited significant improvements solely in the SGDS-K (*p* < 0.05). A comparative analysis of the changes between the two groups demonstrated significant differences in both the SGDS-K and PSS (*p* < 0.05 and *p* < 0.01) (Table 4).

### 3.3. ADL and Social Participation

The analysis of changes in ADL/IADL abilities before and after the intervention indicated no differences in scores for either group, with no statistically significant changes (*p* > 0.05). The assessment of changes in the community integration domain before and after the intervention revealed significant improvements in social integration and total scores within the experimental group (*p* < 0.05). In contrast, no significant changes were observed across any domains within the control group. A comparison of the changes between the two groups revealed a more substantial improvement in social integration within the experimental group, with statistically significant differences (*p* < 0.05) (Table 5).

## 4. Discussion

This study is aimed at investigating the impact of client-centered occupational therapy intervention on the mental health, ADL, and community participation of older adults with depression. Participants were selected as individuals over 65 years of age who had been diagnosed with depression for at least 2 years. The participants were randomly assigned to either the experimental group (*n* = 15) or the experimental group (*n* = 15). The client-centered occupational therapy was conducted for the experimental group, while the case management and a silver gymnastics program were provided for the control group.

To establish the client-centered occupational therapy intervention, we utilized CCSCI developed by Guidetti et al. [21], adapting it as needed to align with the purpose of this study. Personal goal activities were identified using the COPM, while the BTC was employed to identify unproductive periods before establishing plans for the targeted activities. At the beginning of each session, a review of the activities carried out in the preceding session took place, with checks on activity performance conducted as required. The control group received encouragement and monitoring to ensure ongoing treatment adherence. This was complemented by case management services that provided assistance with scheduling hospital visits and facilitated participation in a silver gymnastics program developed by the National Health Insurance Service.

The study results showed that both the experimental and control groups experienced a reduction in depression following their respective interventions. Kim and Kang [18] observed a significant decrease in symptoms of depression after a 4-week client-centered occupational therapy intervention for patients with chronic schizophrenia. In a similar vein, Choi and Won [33] discovered that case management activities, which were provided through home visits for older adults with depression living alone, effectively reduced their symptoms of depression. These two studies show that reductions in depression can be achieved not only through client-centered occupational therapy interventions but also through case management activities. While both groups exhibited reductions in depression, a significant diminishment in anxiety and stress was observed in the experimental group only. Anxiety often emerges in the face of unfamiliar or new situations or in the presence of unmet psychological needs in situations where fundamental human needs are under threat. Patients with depression may also feel anxious due to apprehensions about unrealistic dangers or adverse future events [30]. This study suggests that the administration of BTC to the experimental group may have contributed to diminishing anxiety by aiding participants in recognizing their values and priorities and by structuring daily routines thus making everyday life predictable. Furthermore, the engagement of the experimental group in client-centered activities, including handicrafts and gardening, presumably contributed to the reduction of anxiety and stress. Caddy, Crawford, and Page [31] reported that the incorporation of creative activities into occupational therapy for psychiatric patients reduced anxiety. Likewise, research by Walsh, Martin, and Schmidt [34] showed that engaging in creative handicraft activities effectively reduced stress. Lim, Lee, and Yang [35] further reported that creative handicraft activities, when applied therapeutically, can efficiently alleviate stress. Similarly, Yun and Choi [36] observed that a horticultural therapy program significantly contributed to reducing stress among older adults with dementia, thus aligning with the findings of this study.

Analysis of changes in ADL/IADL skills before and after interventions in both experimental and control groups showed no change in scores for either group nor were there any statistically significant differences observed. Previous research has indicated that, compared to adults, the older adults face more challenges, including physical illnesses, financial difficulties, living alone, and social isolation. Among these, physical function status is a critical determinant of ADL disability. It has been shown that the ADL skills of older adults with depression are more significantly affected by their physical function status than by depression symptoms. This suggests that improvements in ADL skills may rely more heavily on treating physical illnesses than on addressing depression itself [37, 38]. In this study, neither the client-centered occupational therapy provided to the experimental group nor the case management and silver gymnastics offered to the control group significantly improved participants' physical functions. The activities chosen by participants, such as gardening, handicrafts, and cognitive exercises for dementia prevention, were predominantly sedentary activities rather than physically engaging activities for body coordination. For future research focused on improving ADL skills among older adults, it may be important to balance respecting participants' activity preferences with selecting activities that can effectively enhance physical function.

Analysis of the changes in community participation of the experimental and control groups revealed significant improvement in the score for social integration and the total score in the experimental group, whereas the control group did not show significant changes in any of the assessment items. Zacher [39] mentioned the necessity of environmental control for older adults to enable them to spend their time effectively and meaningfully. Kim and Jeon [40] reported that the amount of leisure activities increased among older adults after participating in a lifestyle redesign program, emphasizing the importance of adjusting the living environment for the older adults. In this study, by identifying the daily life patterns of the participants and then inducing them to engage in activities that reflect their needs during their otherwise meaningless time, these make it possible for them to lead a more meaningful and regular daily life and participate in social activities, which resulted in an overall improvement in community participation functions.

A comparison of the differences in changes between the experimental and control groups after the intervention revealed significant differences in depression, stress, and the social integration domain of community integration but no significant between-group differences in changes in anxiety, ADL skills, and the domains of community integration other than social integration. The significant improvements in depression, stress, and social integration observed within the experimental group serve as strong indicators of the effectiveness of client-centered occupational therapy. Numerous studies have shown that client-centered occupational therapy effectively alleviates depression, while activities such as handicrafts and gardening reduce stress, and home visit occupational therapy enhances social participation [18, 41, 42]. Significant improvement was also observed in the depression for both groups, with the extent of improvement statistically more significant in the experimental group. Jeong, Shin, and Yang [43] noted that job satisfaction is lower in elderly patients with depression compared to the general older adults population, attributing it to their reduced internal motivation. They proposed that assisting individuals in engaging with their environment, tailored to their personal characteristics during daily activities, and fostering motivation through activity-based engagement may contribute to a decrease in their depression levels. Additionally, Kim and Kang [18] stated that client-centered occupational therapy interventions that actively reflect the client's needs help to reduce depression in patients with psychiatric issues and are effective in inducing active participation in activities, supporting the findings of this study.

Despite the significance of this study for reaffirming the positive effects of client-centered occupational therapy interventions on depression, anxiety, stress, and social integration among older adults with depression, it has several limitations. Firstly, the small sample size limits the generalizability of the results to the broader population of older adults with depression. Secondly, since the participants of this study are older adults, it is impossible to completely exclude the impact of physical illnesses or conditions at the time of assessment. Third, assessment tools developed in Korea, which are not used internationally, were used to measure ADL and social partitioning. Additionally, no follow-up tests were conducted to confirm the lasting effects of the interventions. Future research should overcome these issues by recruiting more participants, ensuring consistent health status before and after the evaluations, and carrying out follow-up tests to ascertain the persistence of the intervention effects. In addition, in order to give greater meaning to the results, it would be better to use an internationally recognized assessment tool in the future. Despite these limitations, there were few cases of applying client-centered occupational therapy intervention to depression patients, and in the absence of community participation studies on older adults with depression, this study suggests that occupational therapy intervention can be a useful intervention method for older adults with depression.

## 5. Conclusion

This study showed that client-centered occupational therapy interventions effectively reduce depression, anxiety, and stress among older adults with depression, while also enhancing community engagement. Given the expanding role of occupational therapists in the mental health sector, the findings of this study are expected to serve as valuable guidance for occupational therapists working with psychiatric disorders.

## Figures and Tables

**Table 1:** Client-centered self-care intervention (CCSCI).

**Stage**	**Description**
1	(Therapist) Establish rapport with the client
2	(Therapist) Analyze the client's occupational performance by observing and analyzing their activities
3	(Therapist) Through evaluation, identify challenges within the client's activities
4	(Therapist) Guide the client in setting three performance goals using the COPM
5	(Client) Develop a plan for executing the goals
6	(Therapist) Assist the client in refining the performance plan for greater success. Encourage the client to write an activity log post-activity to foster accountability for their goals and performance. This log can serve as a documented resource for performance training
7	(Therapist) Communicate the client's goals and performance plan with the broader rehabilitation team
8	(Client and therapist) Implement activities as per the devised plan
9	(Client and therapist) Evaluate previous performance strategies and set new goal

**Table 2:** Client-centered occupational therapy process according to CCSCI.

**Session**	**Activities**	**CCSCI**
1	Building rapport with the client	Stage 1
	Performing pre-evaluation: SGDS-K, STAI-KYZ, PSS, K-ADL, K-IADL, K-CIQ	Stage 3
2	Setting the goals COPM, BTC	Stage 4
	Observing and analyzing activities	Stage 2
3	Setting up a plan for occupational performance	Stage 5
4-15	Adjusting the planned activities	Stage 6
	Performing the planned activities	Stage 8
16	Performing postevaluation: SGDS-K, STAI-KYZ, PSS, K-ADL, K-IADL, K-CIQ	Stage 9

**Table 3:** Demographics and baseline characteristics.

**Variable**	**Experiment group (** **n** = 15**)**	**Control group (** **n** = 15**)**	**p**
Age (years)^a,c^	75.07 (8.18)	75.80 (6.37)	0.966
Gender, *n* (%)^b,e^			0.700
Male	6 (40.0)	4 (26.7)	
Female	9 (60.0)	11 (73.3)	
Educations (years)^a,c^	9.67 (3.18)	5.80 (5.83)	0.104
Occupation, *n* (%)^b,d^			0.487
Unemployed	11 (73.3)	9 (60.0)	
Agriculture	2 (13.3)	2 (13.3)	
Senior employment scheme	0 (0.0)	1 (6.7)	
Sales/service	2 (13.3)	2 (13.3)	
Living arrangement, *n* (%)^b,d^			0.430
Living alone	9 (60.0)	7 (46.7)	
Living with spouse	5 (33.3)	6 (40.0)	
Living with spouse/children	1 (6.7)	2 (13.3)	
Baseline characteristics^a,c^			
SGDS-K	7.93 (2.18)	8.20 (1.27)	0.916
STAI-KYZ(S)	43.87 (8.27)	44.87 (6.56)	0.950
STAI-KYZ(T)	42.53 (5.30)	40.47 (5.18)	0.183
PSS	19.60 (4.47)	19.53 (4.64)	0.586	
K-ADL	7.53 (0.91)	7.13 (0.35)	0.169	
K-IALD	12.40 (3.54)	12.33 (3.75)	0.690	
K-CIQ (home integration)	7.40 (2.58)	8.13 (2.26)	0.352	
K-CIQ (social integration)	6.73 (1.48)	5.60 (2.20)	0.127	
K-CIQ (productive activity)	3.07 (1.53)	2.60 (2.35)	0.047⁣^∗^	
K-CIQ (total)	17.20 (4.09)	16.33 (4.42)	0.616	

^a^Data are displayed as mean (SD).

^b^Data are displayed as numbers (percentage).

^c^A significant difference between the two groups using the Mann–Whitney *U* test.

^d^A significant difference between the two groups using the *χ*^2^ test.

^e^A significant difference between the two groups using Fisher's exact test.

⁣^∗^*p* < 0.05.

**Table 4:** Comparison of depression, anxiety, and stress between pretest and posttest and the variance between control and experimental groups.

		**Pretest**	**Posttest**	**Z**	**p** ^**b**^	**Between groups**	**Z**	**p** ^**c**^
		**M** ** (SD)**	**M** ** (SD)**			**M** ** (SD)**		
SGDS-K	E	7.93 (2.19)^a^	5.73 (2.58)	−3.055	0.002⁣^∗∗^	−2.20 (1.74)	−2.076	0.38⁣^∗^
	C	8.20 (1.27)	7.27 (1.39)	−2.507	0.012⁣^∗^	−0.93 (1.16)		
STAI-KYZ(S)	E	43.87 (8.27)	40.73 (6.68)	−1.995	0.046⁣^∗^	−3.13 (5.51)	−1.392	0.164
	C	44.87 (6.56)	44.40 (6.72)	−0.420	0.674	−0.46 (4.78)		
STAI-KYZ(T)	E	42.53 (5.30)	39.53 (3.70)	−2.398	0.016⁣^∗^	−3.00 (4.02)	−0.313	0.755
	C	40.47 (5.18)	39.47 (4.84)	−1.620	0.105	−1.00 (2.26)		
PSS	E	19.60 (4.47)	15.13 (4.67)	−2.393	0.017⁣^∗^	−4.47 (5.01)	−0.273	0.006⁣^∗∗^
	C	19.53 (4.64)	19.60 (2.44)	−0.179	0.858	0.06 (2.49)		

Abbreviations: C, control group; E, experimental group.

^a^Data are displayed as mean (SD).

^b^A significant difference from baseline after intervention in each group using the Wilcoxon signed-rank test.

^c^A significant difference between the two groups using the Mann–Whitney *U* test.

⁣^∗^*p* < 0.05.

⁣^∗∗^*p* < 0.01.

**Table 5:** Comparison of activities of daily living and social participation between pretest and posttest and the variance between control and experimental groups.

		**Pretest**	**Posttest**	**Z**	**p** ^b^	**Between groups**	**Z**	**p** ^c^
		**M** ** (SD)**	**M** ** (SD)**			**M** ** (SD)**		
K-ADL	E	7.53 (0.92)^a^	7.53 (0.92)	0.000	1.000	0	0	1.000
	C	7.13 (0.35)	7.13 (0.35)	0.000	1.000	0		
K-IADL	E	12.40 (3.54)	12.40 (3.54)	0.000	1.000	0	0	1.000
	C	12.33 (3.74)	12.33 (3.74)	0.000	1.000	0		
K-CIQ (home integration)	E	7.40 (2.59)	7.40 (2.59)	0.000	1.000	0	0	1.000
	C	8.13 (2.26)	8.13 (2.26)	0.000	1.000	0		
K-CIQ (social integration)	E	6.73 (1.49)	7.20 (1.32)	−2.070	0.038⁣^∗^	0.46 (0.74)	−2.035	0.042⁣^∗^
	C	5.60 (2.20)	5.67 (2.02)	−1.000	0.317	0.06 (0.26)		
K-CIQ (productive activity)	E	3.07 (1.53)	3.07 (1.53)	0.000	1.000	0	−1.258	0.199
	C	2.60 (2.35)	2.93 (2.34)	−1.342	0.180	0.33 (1.05)		
K-CIQ (total)	E	17.20 (4.09)	17.80 (4.49)	−2.060	0.039⁣^∗^	0.60 (1.12)	−0.543	0.587
	C	16.33 (4.42)	16.73 (4.40)	−1.342	0.180	0.40 (1.12)		

Abbreviations: C, control group; E, experimental group.

^a^Data are displayed as mean (SD).

^b^A significant difference from baseline after intervention in each group using the Wilcoxon signed-rank test.

^c^A significant difference between the two groups using the Mann–Whitney *U* test.

⁣^∗^*p* < 0.05.

⁣^∗∗^*p* < 0.01.

## Data Availability

The data used to support this study's findings are available from the corresponding author upon request.
